# Anticancer properties of cannabidiol and Δ^9^-tetrahydrocannabinol and synergistic effects with gemcitabine and cisplatin in bladder cancer cell lines

**DOI:** 10.1186/s42238-023-00174-z

**Published:** 2023-03-04

**Authors:** Erin G. Whynot, Andrea M. Tomko, Denis J. Dupré

**Affiliations:** Faculty of Medicine, Department of Pharmacology, Dalhousie University, PO BOX 15 000, 5850 College St., Sir Charles Tupper Medical Building, Halifax, NS B3H 4R2 Canada

**Keywords:** Bladder cancer, Gemcitabine, Cisplatin, Cannabidiol, Δ^9^-Tetrahydrocannabinol, Cannabichromene, Cannabivarin, Synergy

## Abstract

**Introduction:**

With the legalization of cannabis in multiple jurisdictions throughout the world, a larger proportion of the population consumes cannabis. Several studies have demonstrated anti-tumor effects of components present in cannabis in different models. Unfortunately, little is known about the potential anti-tumoral effects of cannabinoids in bladder cancer and how cannabinoids could potentially synergize with chemotherapeutic agents. Our study aims to identify whether a combination of cannabinoids, like cannabidiol and Δ^9^-tetrahydrocannabinol, with agents commonly used to treat bladder cancer, such as gemcitabine and cisplatin, can produce desirable synergistic effects. We also evaluated if co-treatment with different cannabinoids resulted in synergistic effects.

**Methods:**

We generated concentration curves with several drugs, including several cannabinoids, to identify the range at which they could exert anti-tumor effects in bladder cancer cell lines. We tested the cytotoxic effects of gemcitabine (up to 100 nM), cisplatin (up to 100 μM), and cannabinoids (up to 10 μM) in T24 and TCCSUP cells. We also evaluated the activation of the apoptotic cascade and whether cannabinoids have the ability to reduce invasion in T24 cells.

**Results:**

Cannabidiol, Δ^9^-tetrahydrocannabinol, cannabichromene, and cannabivarin reduce cell viability of bladder cancer cell lines, and their combination with gemcitabine or cisplatin may induce differential responses, from antagonistic to additive and synergistic effects, depending on the concentrations used. Cannabidiol and Δ^9^-tetrahydrocannabinol were also shown to induce apoptosis via caspase-3 cleavage and reduce invasion in a Matrigel assay. Cannabidiol and Δ^9^-tetrahydrocannabinol also display synergistic properties with other cannabinoids like cannabichromene or cannabivarin, although individual cannabinoids may be sufficient to reduce cell viability of bladder cancer cell lines.

**Discussion:**

Our results indicate that cannabinoids can reduce human bladder transitional cell carcinoma cell viability, and that they can potentially exert synergistic effects when combined with other agents. Our in vitro results will form the basis for future studies in vivo and in clinical trials for the development of new therapies that could be beneficial for the treatment of bladder cancer in the future.

**Supplementary Information:**

The online version contains supplementary material available at 10.1186/s42238-023-00174-z.

## Introduction

The most frequently diagnosed bladder cancer is transitional cell carcinoma (TCC), accounting for more than 90% of all bladder cancers (Pons et al. 2011). Lower grade, superficial non-muscle invasive tumors account for the majority of newly diagnosed TCC; however, most tumors will recur in patients with worsening grade and stage (Bellmunt et al. 2020). Without treatment, the median survival time before the development of effective chemotherapy rarely exceeded 3 to 6 months, but advances in combination chemotherapy have increased median survival times to 14 months. Systemic combination chemotherapy, such as the methotrexate, vinblastine, doxorubicin, and cisplatin (MVAC) regimen, has proven activity in advanced bladder cancer, but significant toxicity is observed with a treatment-related mortality of about 4%, and some patients are not eligible to receive cisplatin chemotherapy (Chester et al. 2004; Li et al. 2005). Thus, there is a need for alternative therapies that provide improved survival outcomes or similar survival benefits with reduced toxicity compared to the MVAC regimen. Gemcitabine-based therapy can be administered as intravesical instillations with minimal bladder irritation, as well as systemically (Moore et al. 1997; Laufer et al. 2003). Gemcitabine-cisplatin combination therapy is effective and safe and is frequently used as first-line therapy against metastatic bladder cancer (Moore et al. 1999; von der Maase et al. 2000; Bellmunt et al. 2012). While the toxicity profile has been improved using this combination, the efficacy of the treatment remains relatively similar to treatment with the MVAC regimen.

Tobacco smoking is one of the most important risk factors for the development of bladder cancer and is associated with a 2- to six fold increase in the lifetime risk of urothelial cancer (Boffetta 2008; Freedman et al. 2011). Several studies have noted that a significant proportion of tobacco smokers also use cannabis. A study on the effects of cannabis and/or tobacco use was performed where men were followed over an 11-year period. Consumption of tobacco only was associated with an increased risk of bladder cancer (hazard regression [HR], 1.52), whereas cannabis use alone was associated with a 45% reduction in bladder cancer incidence (*HR*, 0.55). Using both cannabis and tobacco was associated with an intermediate HR of 1.28 (Thomas et al. 2015). The metabolism of cannabis reveals that 65% of cannabis is excreted in the feces and 20% in urine (Lemberger et al. 1971). Chronic cannabis use causes accumulation of Δ^9^-tetrahydrocannabinol (THC) and its metabolite 11 nor-9 carboxy-Δ^9^-tetrahydrocannabinol (THC-COOH) in adipose tissue such that it is excreted into the urine for as long as 30 to 60 days from the time chronic use is halted. THC and THC-COOH can be found in urine at levels greater than 500 ng/mL (around 1.6 μM) for chronic and/or recent cannabis users.

Over 100 phytocannabinoids have been identified (Mehmedic et al. 2010), but Δ^9^-tetrahydrocannabinol is the most common cannabinoid produced in the *Cannabis*plant (de Meijer et al. 2003). Cannabidiol (CBD) is the most common cannabinoid in hemp and second most prevalent in the majority of cannabis cultivars, with a versatile pharmacological profile (Mechoulam 2005). Interestingly, studies have found that cannabinoids inhibit tumor cell growth and induce apoptosis in various cancer cells (Blázquez et al. 2006; Blázquez et al. 2008; Guzmán et al. 2006; Carracedo et al. 2006; Javid et al. 2016; Blasco-Benito et al. 2018; Tomko et al. 2019). Despite the use of cannabis in the population and evidence of anti-tumoral activity by cannabinoids, little is known about the anticancer effects of cannabis use in bladder cancer. Recently, a study suggested that cannabis-derived cannabichromene (CBC) and Δ^9^-tetrahydrocannabinol displayed some synergy when used together in a model of urothelial cell carcinoma (Anis et al. 2021). Further research is required to understand the effect of the numerous compounds present in cannabis to understand which exert the best anti-tumoral effects and how they may affect current chemotherapeutic agents. Our study presents the results of our investigation of the effects of Δ^9^-tetrahydrocannabinol and cannabidiol alone or in the presence of other cannabinoids, gemcitabine, cisplatin, or the combination of cisplatin and gemcitabine together in bladder cancer cell lines.

## Materials and methods

### Drugs

Gemcitabine, cisplatin, Δ^9^-tetrahydrocannabinol, and cannabidiol were obtained from Millipore-Sigma. Cannabichromene, cannabivarin, rimonabant, *SR* 144,528, and A-967079 were obtained from Cayman Chemical.

### Cell culture

Human bladder transitional cell carcinoma T24 (ATCC® HTB4™), TCCSUP (ATCC® HTB5™), and non-tumorigenic human bladder epithelial cells HBlEpC (938-05a) (Cell Applications Inc.) were cultured in McCoy’s 5A and Eagle’s Minimum Essential Medium (Millipore-Sigma), respectively, with 1% penicillin–streptomycin containing 10% fetal bovine serum (Gibco, Life Technologies) at 37 °C, in a 5% CO_2_atmosphere. It was demonstrated that in vitro models can adequately reproduce clinically relevant results and may be suitable to identify novel substances for the treatment of bladder cancer (Vallo et al. 2015).

### Cytotoxicity assays

T24, TCCSUP, and HBlEpC cells were seeded at 3000 cells/well in 96-well plates and grown for 24 h before adding drugs. Cells were treated with increasing concentrations of various drugs for 48 h. To assess viability, alamarBlue® (Bio-Rad Laboratories) was added to each well and incubated for 4 h at 37 °C as per the manufacturer’s instructions. Fluorescence was measured following excitation at 540 nm, and emission was read at 590 nm with a Biotek Cytation 3. Data are expressed as the percentage of viable cells vs. vehicle-treated cells, normalized as 100% and represented as mean ± SEM. Experiments using antagonists used the same methodology with the addition of the antagonist at 2 × its reported *IC*_50_ value, incubated in presence of the cannabinoids tested, for 24 h. The *p*-values represent data from at least three independent experiments.

### Cell lysis and Western blotting

T24 cells were lysed with RIPA buffer (150 mM NaCl, 50 mM Tris–HCl pH 7.5, 1% NP4O, 0.5% sodium deoxycholate, 0.1% sodium dodecyl sulfate, and 1 complete EDTA-free protease inhibitor cocktail tablet (Roche). BSA-coated beads (Protein A-Sepharose, Sigma-Aldrich) and 10% DNase I (Sigma-Aldrich) were added to remove nucleic acid and organellar material from the sample. Lysates were mixed 50:50 with 2 × Laemmli buffer and 2-mercaptoethanol (Bio-Rad Laboratories). Samples were run on a SDS–PAGE gel and transferred to nitrocellulose membranes before being blocked in a 10% skim milk powder/PBS solution for 60 min and incubated overnight at 4 °C with their respective primary antibodies (cleaved Caspase 3 (p11): sc-271759 from Santa Cruz Biotechnologies). Chemiluminescence was performed on nitrocellulose membranes using Western Lightning® Plus-ECL Enhanced Chemiluminescence Substrate (PerkinElmer) before exposing them to X-ray film and development.

### Apoptosis assay

T24 cells were grown on glass coverslips in 6-well plates and then treated with methanol or 2.5 µM cannabinoids for 24 h. The Annexin V apoptosis detection kit (Santa Cruz Biotechnologies) was used to determine the rate of apoptosis. Cells were harvested and washed with PBS and then resuspended in Annexin V Assay Buffer following the manufacturer’s instructions. Cells were gently shaken in the dark with propidium iodide (PI) and Annexin V-FITC-conjugated stain for 20 min. Cells were then examined by fluorescence microscopy, and at least 5 fields of view were recorded using an Olympus IX81 microscope equipped with a Photometrics coolSNAP HQ2 camera and an X-cite series 120Q light source. Annexin V stain was excited at 488 nm, and images were captured at 525 nm. PI was excited at 535 nm, and images were captured at 617 nm. Rates of early apoptosis were determined by dividing the number of cells that stained positive for Annexin-V divided by the total number of cells (Martin et al. 2019; Young et al. 2015).

### Transwell migration

T24 cells were suspended in McCoy’s 5A medium with no FBS at a concentration of 150,000 cells/mL. Two-hundred and fifty microliters of 0.2% FBS medium containing the vehicle control was added into the top portion of a transwell migration well that contains a polycarbonate membrane (Costar). Two-hundred and fifty microliters of the T24 cell suspension was also added to the top portion of the migration well. In the bottom portion of the well, 700 µL of McCoy’s 5A medium containing 10% FBS was added to direct migration. Cells were incubated at 37 °C under these conditions for 24 h. Following incubation, media and cells that did not migrate were removed with a dampened cotton swab. Cells were then fixed in methanol for 10 min and stained with 3.5 g/L crystal violet in 2% ethanol for 10 min. Wells were rinsed thoroughly with dH_2_O and left to dry overnight. Cells that migrated were counted with an Olympus CKX41 light microscope. The total number of cells that migrated under vehicle conditions served as 100% for invasion assay calculations.

### Matrigel invasion

Growth factor-reduced 8.0 micron Matrigel Invasion Chambers (Corning) were added to a 24-well plate. Matrigel Invasion Chambers were hydrated for 1 h at 37 °C with 250 µl of McCoy’s 5A medium containing 0.2% FBS penicillin–streptomycin. T24 cells were then seeded in McCoy’s 5A without FBS at a concentration of 150,000 cells/mL. Following hydration, 250 µL of the T24 cell suspension was added to the top portion of each invasion well, with a final cannabinoid concentration of 2.5 µM. Seven-hundred microliters of McCoy’s 5A containing 10% FBS was added to the bottom portion of each well. Invasion wells were incubated at 37 °C for 24 h. After 24 h, media and cells that did not invade were removed from the inside of the well with a dampened cotton swab. Wells were placed in methanol for 10 min and then transferred into a 3.5 g/L crystal violet in 2% ethanol solution for 10 min. Wells were then rinsed with dH_2_0 and left to dry overnight. Cells that invaded through the Matrigel were counted using an Olympus CKX41 light microscope. Percent invasion was calculated by dividing the number of cells invaded in each condition by the number of cells that migrated in the control.

### Assessment of synergism, additivity, or antagonism

Synergies between Δ^9^-tetrahydrocannabinol or cannabidiol and gemcitabine, cisplatin, or a combination of gemcitabine/cisplatin were studied using the checkerboard assay in T24 cells. Synergy was also assessed between Δ^9^-tetrahydrocannabinol or cannabidiol and cannabivarin or cannabichromene. Briefly, the synergy assay was performed with 3000 cells in 96-well plates with a final volume of 100 μL per well. Cannabinoid concentrations ranged from 0 to 10 mM and gemcitabine and cisplatin concentrations between 0 and 100 mM. Fluorescence was quantified as described before using alamar Blue**® **after 48-h treatment. The analysis was performed using SynergyFinder 2.0 (Ianevski et al. 2020), where the Bliss independence drug interaction model was used. A synergy score of <  − 10 was considered as antagonistic, a range from − 10 to + 10 as additive, and >  + 10 as synergistic (Ianevski et al. 2020a; 2020b). Drug combination responses were also plotted as concentration–response curves using GraphPad Prism software and were used to determine statically significant and synergistic combinations.

### Statistical analysis

Statistical analysis was completed using GraphPad Prism software. All error bars are representative of mean ± SEM. Unpaired Student’s *t*-tests were performed for analysis of two independent groups. One-way ANOVA with Tukey’s post hoc test was used to assess multigroup comparisons. *p*-values are reported as follows: *p < 0.05, **p < 0.01, and ***p < 0.001.

## Results

### Effect of individual drugs on cell viability

**Fig. 1:**
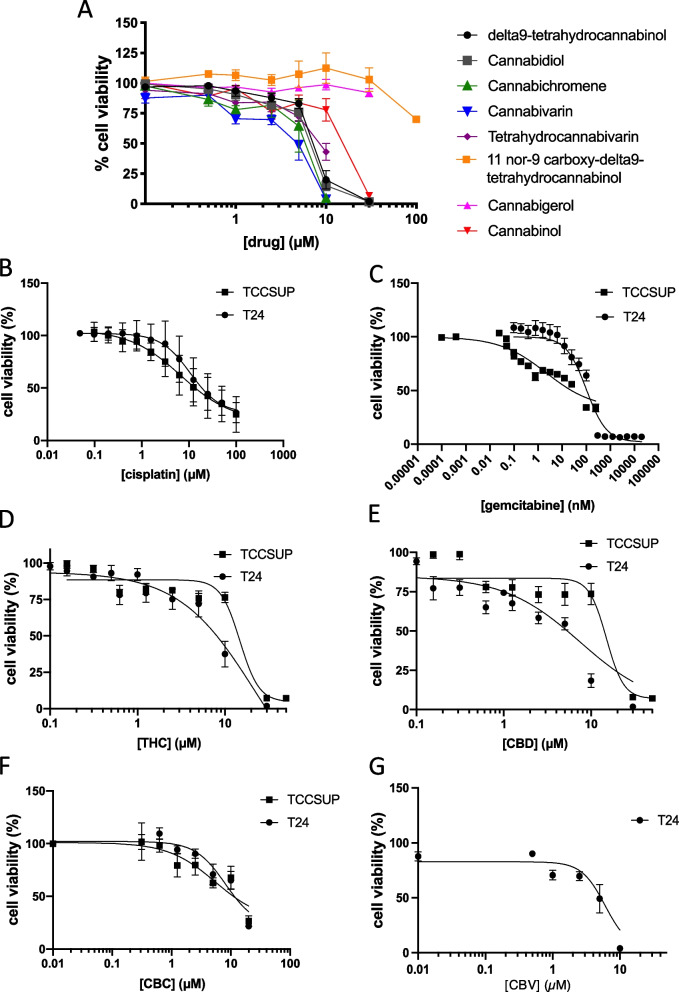
Effects of individual drugs on cell viability. Cell viability of T24 and TCCSUP cells was assessed after 48-h treatment with various drugs in a concentration-response experiment. **A** All cannabinoids tested in the study in T24 cells are compared in the same graph. **B** Cisplatin alone. **C** Gemcitabine alone. **D** Δ^9^-Tetrahydrocannabinol alone. **E** Cannabidiol alone. **F** Cannabichromene alone. **G** Cannabivarin alone, in the indicated cell lines. Results are means ± SEM of at least 3 independent experiments

Several cannabinoids were tested for the effect on the cell viability of two commonly studied bladder cancer cell lines, T24 and TCCSUP (Zuiverloon et al. 2018). Figure1 A shows the various cannabinoids tested in our study, in comparison with each other in T24 cells. We first evaluated the effects of common chemotherapeutic agents for bladder cancer; cisplatin was cytotoxic to T24 cells and TCCSUP transitional cell carcinoma cells with *EC*_50_ values of 10.75 mM and 6.75 mM, respectively, after 48 h (Fig. 1B). Gemcitabine also displayed cytotoxic activity against the T24 cells with an *EC*_50_ of 102 nM, while the response against the TCCSUP cells was not as strong with an *EC*_50_ of approximately 2 mM (Fig. 1C). *Δ*^9^-Tetrahydrocannabinol yielded *EC*_50_ values of 8.5 mM in T24 cells and 13.5 mM in TCCSUP cells (Fig. 1D), while cannabidiol showed an *EC*_50_ value of 7 mM in T24 cells and 18 mM in TCCSUP cells (Fig. 1E). Among the other cannabinoids tested, cannabivarin and cannabichromene also displayed good reductions in cell viability, with an *EC*_50_ of 5 mM and 6 mM, respectively, in T24 cells (Fig. 1 F and G). In TCCSUP cells, cannabichromene had an *EC*_50_ of 8 µM (Fig. 1F). In HBlEpC cells treated with 10 µM of various cannabinoids, only THCV significantly reduced their cell viability (Suppl. Figure 1). The other cannabinoids evaluated did not significantly reduce the cell viability of the non-tumorigenic bladder cells.

### Effects of blockade of cannabinoid targets on cell viability

**Fig. 2:**
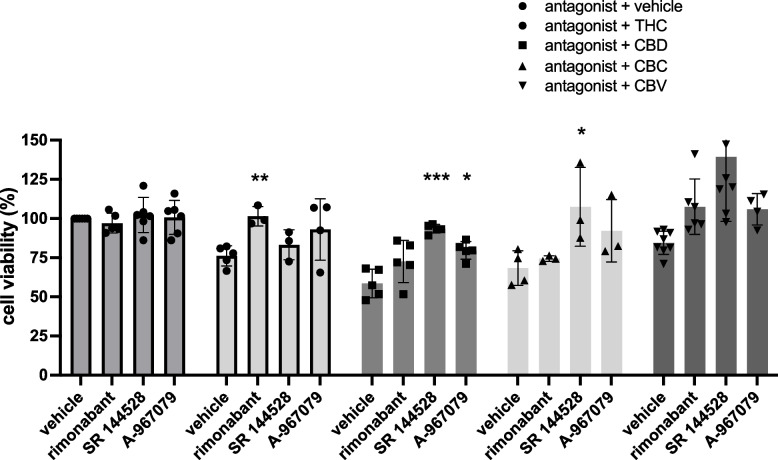
Effects of antagonists on cell viability. Cell viability of T24 cells was assessed after 2-h treatment with various cannabinoids at 5 mM, to identify potential targets by which the cannabinoids mediate their effects on cell viability of bladder cancer cells. The first 4 bars show the effects of the antagonists alone, where their effects were minimal at the concentrations used. The subsequent sets of bars show their effects on blocking Δ.^9^-tetrahydrocannabinol, cannabidiol, cannabichromene, and cannabivarin, respectively. Results are means ± SEM of at least 3 independent experiments. *p < 0.05, ***p < 0.001

The effects of rimonabant (CB1R), *SR* 144,528 (CB2R), and A-967079 (TRPA1) on cell viability of T24 cells were explored to identify potential receptor targets involved in the effects. First, each antagonist did not induce significant reduction in cell viability at the concentration used (20 nM of *SR* 144,528, 13.2 nM of rimonabant, and 134 nM of A-967079) equivalent to 2 × the *IC*_50_ of each compound as reported in the manufacturer’s datasheet/literature (Fig. 2). At 24 h, the 5 mM Δ^9^-tetrahydrocannabinol-induced reduction in cell viability was blocked by rimonabant, suggesting that CB1R is involved in the effects. While not statistically significant in our study, TRPA1 blockade also shown a trend toward improved cell viability. Both CB1R and TRPA1 have previously been shown to bind Δ^9^-tetrahydrocannabinol. The effects of 5 mM cannabidiol were blocked by the CB2R and the TRPA1 antagonists, but without any effect with CB1R antagonism. The effects of 5 mM cannabichromene were also inhibited by the CB2R antagonist.

These results are in accordance with previously identified targets of the various cannabinoids tested. In the case of cannabivarin, little information is available regarding its molecular targets. None of the antagonists significantly blocked the effects of cannabivarin. More experiments are needed to clarify the mechanism of action of cannabivarin and the involvement of specific receptors that mediate the effects.

### Effects of cannabinoids on apoptosis

**Fig. 3:**
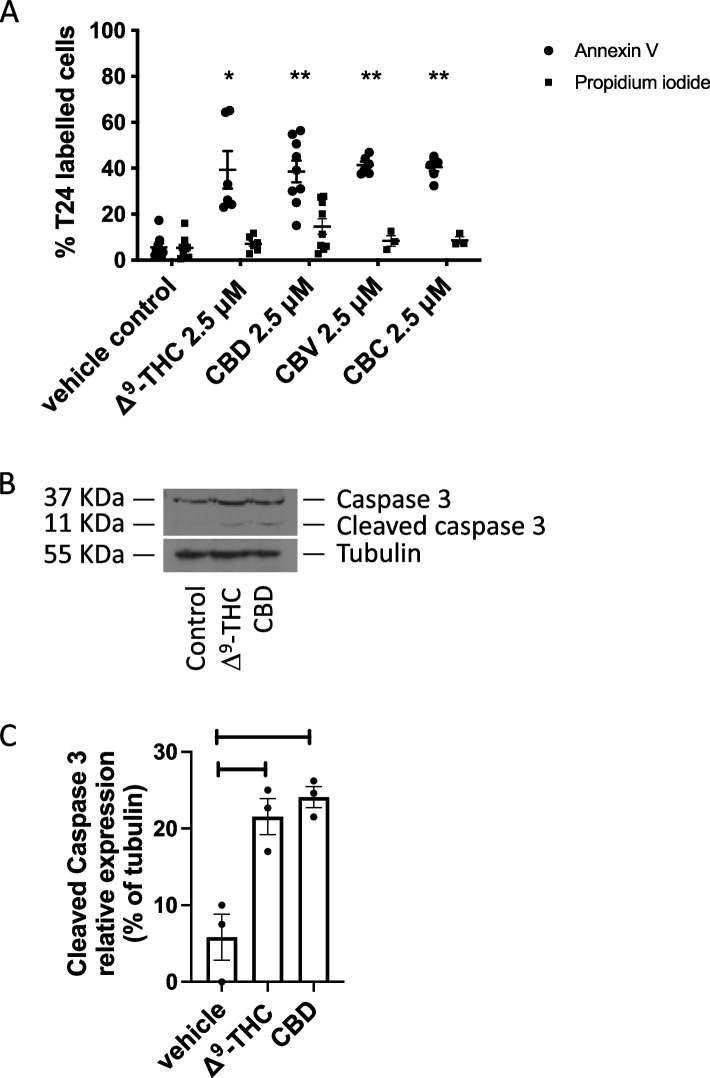
Effects of cannabinoids on apoptosis. T24 cells were treated for 24 h with either the methanol vehicle, Δ^9^-tetrahydrocannabinol, cannabidiol, cannabivarin, or cannabichromene. **A** Histogram showing the % of annexin V-labelled cells and % cells stained for propidium iodide. Cells were counted from three random fields of view on a fluorescence microscope. *p < 0.05, **p < 0.01, *n* = 3. **B** Western blotting analysis was performed using an anti-caspase-3 antibody, and *β*-tubulin was included as a loading control, where a representative blot of *n* = 3 experiments is shown. Cleaved caspase 3 is indicative of activation of this cascade. **C** Quantification of the cleaved caspase 3 relative to tubulin from Western blotting analyses. Results represent the means ± SEM of 3 experiments

It was recently demonstrated in bladder cancer cell lines that cannabidiol and a mixture of Δ^9^-tetrahydrocannabinol and cannabichromene could induce apoptosis (Anis et al. 2021). In the group’s study, no results were shown regarding the effects of Δ^9^-tetrahydrocannabinol alone. Our results confirm the ability of cannabidiol to induce apoptosis (Fig. 3A). Following a 24-h treatment of cells with a concentration of cannabinoid at which we did not detect changes in cell viability (2.5 mM), cannabidiol induced annexin V labelling of 38.5% ± 5.5 in T24 cells, and Δ^9^-tetrahydrocannabinol induced annexin V labelling of 39.3% ± 7.1 in T24 cells. Cannabichromene and cannabivarin at 2.5 mM also induced apoptosis with 40.6% ± 1.6 and 41.4% ± 2.2% of cells labelled with annexin V, respectively. Propidium iodide-labelled cells following cannabinoid treatment did not differ significantly compared to the vehicle control. We investigated the potential involvement of caspase 3 in the induction of apoptosis by the two main cannabinoids Δ^9^-tetrahydrocannabinol and cannabidiol and observed an increase in immunoblotting of cleaved caspase 3 following ligand treatment for 24 h at a concentration of 2.5 mM (Fig. 3 B and C).

### Effects of cannabinoids on invasion

**Fig. 4:**
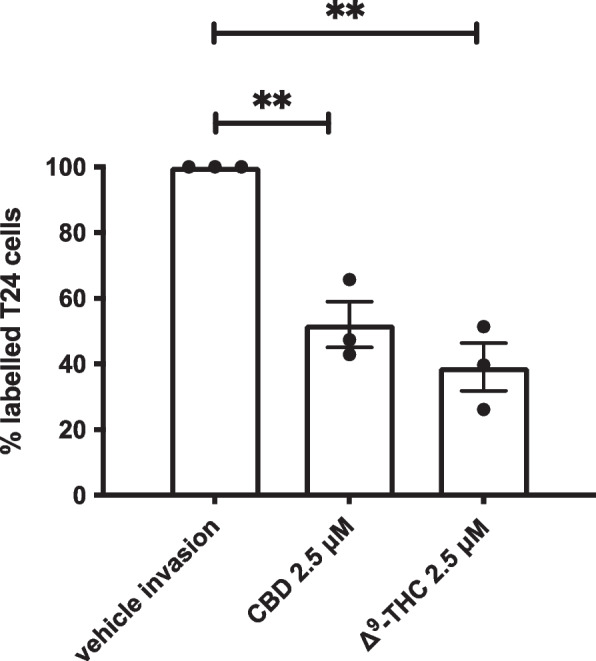
Effects of cannabinoids on invasion. Histogram summarizing Matrigel invasion assays using T24 cells in the presence of either the vehicle control, Δ.^9^-tetrahydrocannabinol, or cannabidiol. Results represent the means ± SEM of 3 experiments. *p < 0.05, ***p < 0.001

In addition to their anticancer effects, we evaluated the potential of Δ^9^-tetrahydrocannabinol and cannabidiol at reducing invasion of the high-grade and invasive T24 cells. An invasion assay was conducted where T24 cells were seeded into Matrigel invasion chambers and treated with the cannabinoids Δ^9^-tetrahydrocannabinol or cannabidiol for 24 h. Our results indicate that T24 cells can invade the Matrigel (Fig. 4). In our control conditions, 25.3% of cells could invade the Matrigel (data not shown) compared to gravity-induced migration. Following treatment of T24 cells with 2.5 µM of Δ^9^-tetrahydrocannabinol for 24 h, only 39.1% of cells could invade the Matrigel. Similarly, treatment of T24 cells with cannabidiol resulted in a reduced fraction of cells (52%) able to invade and cross the Matrigel.

### Assessment of synergy between Δ^9^-tetrahydrocannabinol or cannabidiol and chemotherapeutic agents

**Fig. 5:**
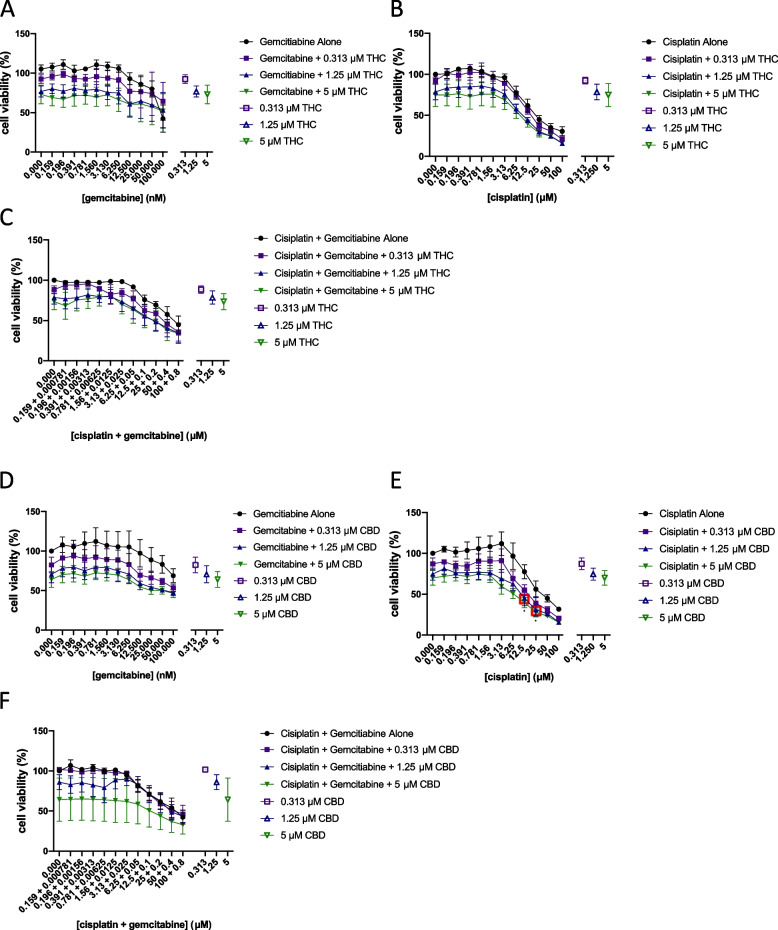
Assessment of synergy between Δ^9^-tetrahydrocannabinol or cannabidiol and chemotherapeutic agents. Concentration-response curves of Δ^9^-THC or CBD combined with different concentrations of chemotherapeutic agents from the matrix are presented in T24 cells. **A** Gemcitabine with selected concentrations of Δ^9^-THC. **B** Cisplatin with selected concentrations of Δ^9^-THC. **C** Gemcitabine:cisplatin with selected concentrations of Δ^9^-THC. **D** Gemcitabine with selected concentrations of CBD. **E** Cisplatin with selected concentrations of CBD. **F** Gemcitabine:cisplatin with selected concentrations of CBD. *X*-axis intervals increase by a value of 2. Hollow points represent each drug’s effects alone. Red boxes indicate the concentrations where combinations of drugs were found to be synergistic as determined by the bliss independence model using SynergyFinder 2.0 software and significantly different from Δ^9^-THC, CBD, or chemotherapeutic treatment alone

**Fig. 6:**
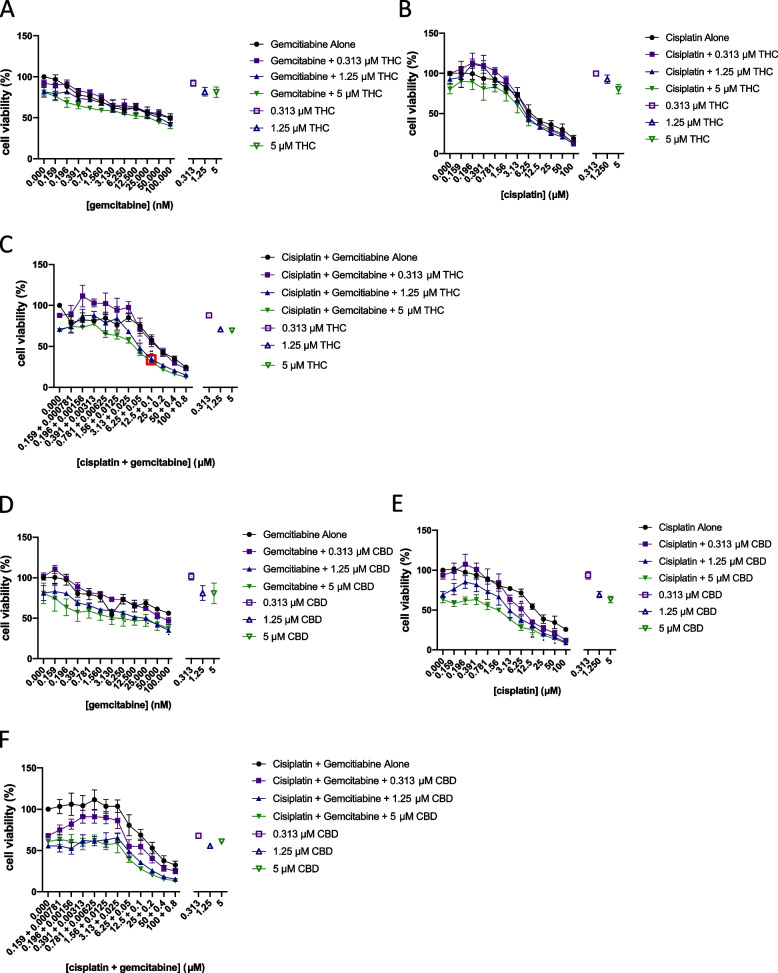
Assessment of synergy between Δ^9^-tetrahydrocannabinol or cannabidiol and chemotherapeutic agents. Concentration-response curves of Δ^9^-THC or CBD combined with different concentrations of chemotherapeutic agents from the matrix cell viability assay are presented in TCCSUP cells. **A** Gemcitabine with selected concentrations of Δ^9^-THC. **B** Cisplatin with selected concentrations of *Δ*^9^-THC. **C** Gemcitabine:cisplatin with selected concentrations of *Δ*^9^-THC. **D** Gemcitabine with selected concentrations of CBD. **E** Cisplatin with selected concentrations of CBD. **F** Gemcitabine:cisplatin with selected concentrations of CBD. *X*-axis intervals increase by a value of 2. Hollow points represent cannabinoid effects alone. Red boxes indicate concentration combinations that are synergistic as determined by the bliss independence model using SynergyFinder2.0 software and that are significantly different from Δ^9^-THC, CBD, or chemotherapeutic treatment alone

It has been suggested that the use of combined gemcitabine-cisplatin treatment displays similar anticancer effects to MVAC, with fewer side effects. Therefore, we decided to test the effects of cannabinoid co-treatment with gemcitabine, cisplatin, or the combination of gemcitabine and cisplatin (GC) on cell viability. The *EC*_50_ for gemcitabine and cisplatin vary from one study to another, but our values are similar to what others have previously found (Mey et al. 2006). Because of the variation in*EC*_50_values, we compared the ratio between gemcitabine and cisplatin among various studies and identified a range of ratios between 1:100 and 1:150 (Ma et al. 2010; Rabenstein et al. 2017). We used a ratio of gemcitabine:cisplatin of 1:125, which is slightly above the *EC*_50_ values we observed (1:105) but in the middle of the range of drug ratios previously published. Figures 5 (T24 cells) and 6 (TCCSUP cells) show concentration–response curves (Ianevski et al. 2020) of several combinations tested. Our results indicate that depending on the concentration of the agents used, a variety of effects can occur, from antagonism to additivity or synergy. Our results indicate some synergy between CBD and cisplatin (synergy score of 14) and some concentrations of CBD with gemcitabine. Some combinations between CBD and cisplatin or gemcitabine resulted in higher levels of antagonism. Δ^9^-Tetrahydrocannabinol displayed synergy with gemcitabine or with the gemcitabine:cisplatin combination with top synergy scores in the 14–17 range. Our results suggest that most of the effects of the combination of cannabinoids with gemcitabine or cisplatin would result in additive effects. Additive effects were observed with cisplatin, as well as some antagonism, depending on the concentrations studied. While some of these values are synergistic, it does not mean necessarily that there is a significant difference physiologically. To assess this, we performed an analysis of the significance of the results between each drug individually and the combination tested. In T24 cells, the combinations of 5 µM CBD with 12.5 and 25 µM cisplatin significantly reduced the cell viability compared to either compound alone and was found to be synergistic (red boxes in Fig. 5E) using the bliss independence model. In TCCSUP cells, the combination of 12.5 or 5 µM Δ^9^-THC with 12.5 µM + 100 nM of cisplatin and gemcitabine respectively significantly reduced the cell viability compared to any of the compounds alone and was found to be synergistic using the bliss independence model (overlapping red boxes in Fig. 6C).

### *Assessment of synergy between Δ*^9^*-tetrahydrocannabinol or cannabidiol and other cannabinoids*

**Fig. 7:**
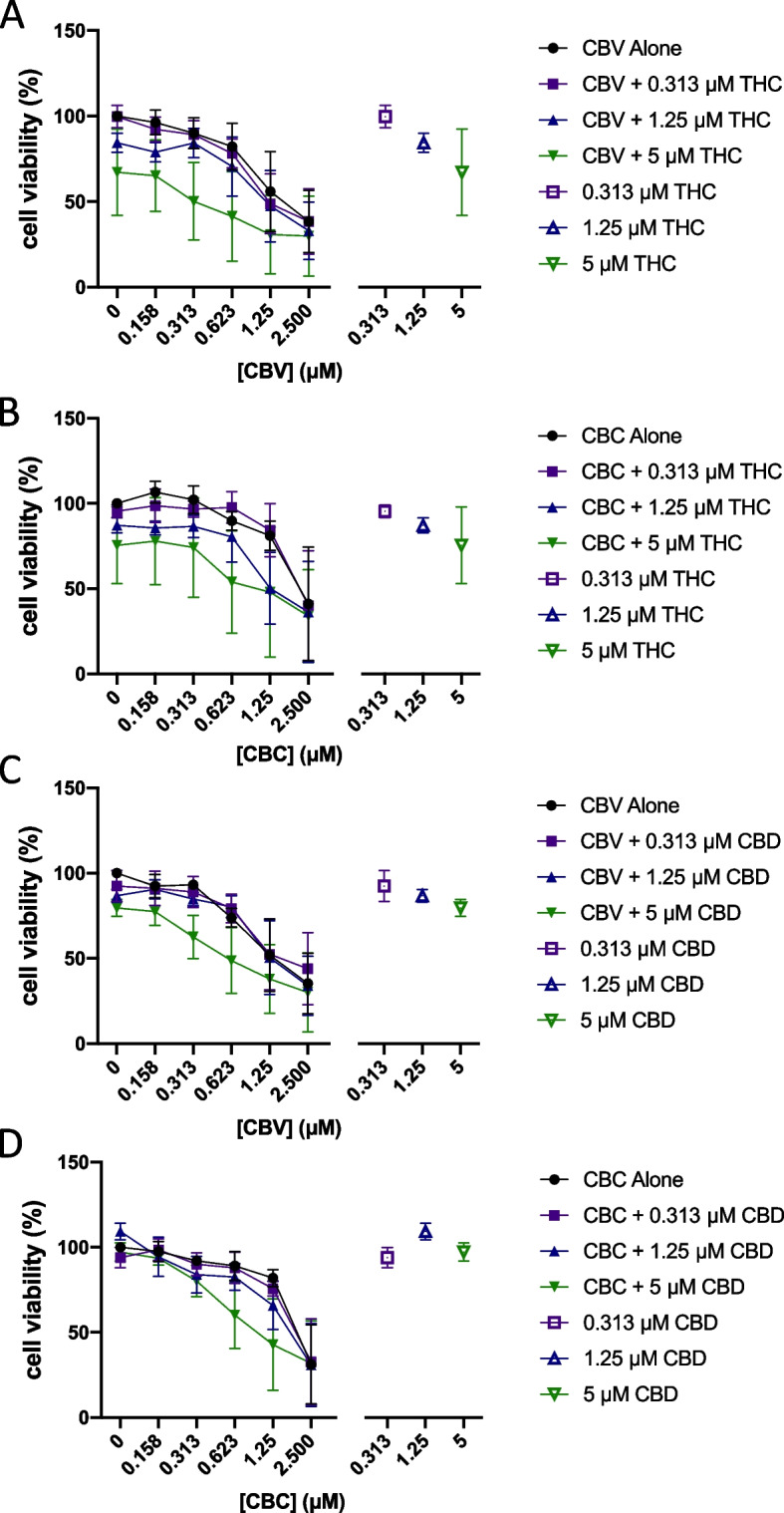
Assessment of synergy between Δ^9^-tetrahydrocannabinol or cannabidiol and other cannabinoids. Concentration-response curves of CBC or CBV combined with different concentrations of Δ^9^-THC or CBD from the matrix cell viability assay are presented in T24 cells. **A** Curve of CBV with selected concentrations of Δ^9^-THC. **B** Curve of CBC with selected concentrations of Δ^9^-THC. **C** Curve of CBV with selected concentrations of CBD. **D** Curve of CBC with selected concentrations of CBD. *X*-axis intervals increase by a value of 2. Hollow points represent cannabinoid effects alone

We tested the effects of the combination of cannabichromene or cannabivarin with low, mid, and higher range concentrations of Δ^9^-tetrahydrocannabinol or cannabidiol, as these four compounds displayed some levels of reduction of cell viability in the bladder cancer cell lines used in this study. Cannabichromene and cannabivarin reduced cell viability with better or similar *EC*_50_ values as cannabidiol in our study. Larger synergy scores were observed with the various cannabinoid combinations. High synergy scores ranging between 38 and 71 were observed for several combinations, in particular the combinations where Δ^9^-THC was present (Fig. 7). Unfortunately, when compared to each drug individually, the synergy of effects was not shown to be significantly different from each drug used individually. This can occur when at least one of the two drugs combined has a large effect, as it is the case here. The combination of cannabidiol and cannabivarin did not produce synergistic effects with the same magnitude as the other cannabinoid combinations tested, with scores in the low synergistic range (between 11 and 23). While the results show synergy when using two cannabinoids, no combination of cannabinoids significantly reduced cell viability compared to the individual compounds alone while demonstrating synergy concurrently using the bliss independence model.

## Discussion

In this study, the effects of several cannabinoids, including Δ^9^-tetrahydrocannabinol and cannabidiol, were tested for their potential anti-tumoral effects in bladder cancer. Our results indicate that cannabinoids can reduce cell viability of human bladder transitional cell carcinoma cell lines. We demonstrate that apoptosis is involved in the process, and that caspase 3 is involved. Additionally, we show that invasion can be diminished by cannabinoids. Finally, we tested the potential synergistic effects of the combination of cannabinoids with current chemotherapeutic treatments or other cannabinoids. Our results show that some synergy occurs with gemcitabine or cisplatin.

Our results indicate variations in the ability of the cannabinoids tested to produce anti-tumoral effects in bladder cancer cell lines. For example, while cannabichromene, cannabivarin, and cannabidiol produced effects that were generally within the same concentration range, cannabigerol, cannabinol, or the metabolite THC-COOH required much larger concentrations to produce an effect. We cannot dismiss that metabolites like THC-COOH could also exert anticancer effects, since they can accumulate at high concentrations in the urine, especially in frequent or heavy cannabis users (Smith-Kielland et al. 1999). Here, we concentrated our investigation on the cannabinoids that displayed the highest levels of anti-tumor activity in vitro, cannabichromene, cannabivarin, cannabidiol, and Δ^9^-tetrahydrocannabinol. The *EC*_50_ values observed were between 5 and 10.5 µM in T24 cells, while *EC*_50_ values were slightly higher in TCCSUP cells. While cannabinoid receptors are expressed in bladder tissue and bladder cancer cells, we did not evaluate the levels of expression of receptors that bind cannabinoids in our study. Variations in expression levels of CB1R and CB2R between the cell lines could contribute to the differences in *EC*_50_ observed between the cell lines, as well as a number of other cellular targets: receptor heteromers comprising one or more cannabinoid receptors; other G protein-coupled receptors or channels being activated by cannabinoids, for example, could potentially explain the µM concentrations needed to display cellular effects in these cell lines versus what would be expected if CB1R or CB2R were solely responsible for the effects. While the µM levels may not be reached when cannabis is consumed, various methods including intravesical therapy, for example, could allow appropriate concentrations of the various cannabinoids to be reached to treat bladder cancer in vivo. When combined with inhibitors of specific receptors, our results revealed that the cannabinoids may be acting through several different receptors in the T24 cells. These results are at least partially consistent with known receptor interactions in the literature. A more detailed analysis of the activation profile of the various potential targets of cannabinoids would be needed to precisely identify by which mechanisms the effects are occurring.

Several reports have indicated that cannabinoids may induce cell death via induction of the apoptotic cascade (Tomko et al. 2020). Our results indicate that apoptosis is induced by cannabinoids, and that caspase 3 is involved, as we detected cleaved caspase 3 following treatment of T24 cells with cannabinoids. These results are similar to what we and others have observed in other cancer types (Tomko et al. 2019; Rieder et al. 2010). Our results also indicate that not only the apoptotic signaling pathways are activated but also other signaling pathways linked to migration and invasion are also altered by cannabinoids. The invasion of high-grade and invasive T24 transitional cell carcinoma cells was reduced following treatment with cannabinoids at a concentration that did not alter cell viability. The results suggest that cannabinoids could potentially be useful to reduce migration and invasion of bladder cancer.

Multiple studies have demonstrated the ability of chemotherapeutic agents used for bladder cancer, like gemcitabine and cisplatin, to act synergistically with other compounds and produce greater anticancer effects (Mey et al. 2006; Ma et al. 2010; Rabenstein et al. 2017). We identified that some concentrations of cannabidiol or Δ^9^-tetrahydrocannabinol acted synergistically with gemcitabine and/or cisplatin. These results remain to be validated in vivo but provide a starting point of the range of concentrations that could be required to generate effects in combination therapy involving cannabinoids.

In recent years, several studies have attempted to characterize how cannabinoids and other compounds present in the cannabis plant work together. Some have suggested that various components of the plant could work together to produce synergistic results. One study investigating the effects of pure cannabinoids versus botanical preparations has shown that their botanical preparation was more potent than pure Δ^9^-THC at producing anti-tumor responses in both in vitro and in vivo breast cancer models (Blasco-Benito et al. 2018). The compounds mediating the effects were not identified. Our group also recently demonstrated that some terpenes can produce synergistic effects with cannabinoids like CBD and Δ^9^-tetrahydrocannabinol (Tomko et al. 2022) in breast cancer cells, and that cannflavin A, a flavonoid unique to cannabis, can produce synergistic effects with cannabinoids, gemcitabine, and/or cisplatin, in bladder cancer cells (Tomko et al. 2022). Again, in bladder cancer, a study demonstrated that the combination of Δ^9^-tetrahydrocannabinol and cannabichromene produces synergistic effects in a bladder cancer model (Anis et al. 2021). We confirmed that CBC and Δ^9^-tetrahydrocannabinol can produce synergistic effects, but our results indicate that the effects were not significantly different from CBC or Δ^9^-tetrahydrocannabinol used individually. We also demonstrated that other cannabinoid combinations induce synergistic effects, as observed for Δ^9^-tetrahydrocannabinol and cannabivarin, as well as cannabidiol and cannabichromene, but again, none of them was significantly different from the effects of the cannabinoids used individually. Our results show the ability of different cannabinoids to produce synergistic effects when combined with other agents like gemcitabine and cisplatin that are significantly different from each drug used alone.

## Conclusions

These results remain to be validated in in vivo models and in human clinical trials, but overall, our results suggest that cannabinoids could potentially be useful in the treatment of bladder cancer. More investigation is needed to determine how they could be used therapeutically in the treatment of cancers, including bladder cancer, whether as single therapy or in combination with other chemotherapeutic agents.

## Supplementary Information

**Additional file 1: Supplemental Fig. 1.** Effects of individual drugs on non-tumorigenic epithelial bladder cells.

## Data Availability

The datasets used and/or analyzed during the current study are available from the corresponding author on reasonable request.

## Abbreviations

TCC: Transitional cell carcinoma
MVAC: Methotrexate, vinblastine sulfate, doxorubicin hydrochloride (Adriamycin), and cisplatin
HR: Hazard regression
THC: Tetrahydrocannabinol
THC-COOH: 11 Nor-9 carboxy-Δ^9^-tetrahydrocannabinol
CBD: Cannabidiol
CBC: Cannabichromene
GC: Gemcitabine and cisplatin
