# Supplementary material for: Anticancer properties of cannabidiol and Δ9-tetrahydrocannabinol and synergistic effects with gemcitabine and cisplatin in bladder cancer cell lines
Source: J Cannabis Res. 2023 Mar 4;5:7. doi: 10.1186/s42238-023-00174-z (PMC9985258; doi:10.1186/s42238-023-00174-z)
Supplement: Supplementary file 1 — Additional file 1: Supplemental Fig. 1. Effects of individual drugs on non-tumorigenic epithelial bladder cells. [file 42238_2023_174_MOESM1_ESM.pptx]

## Slide 1
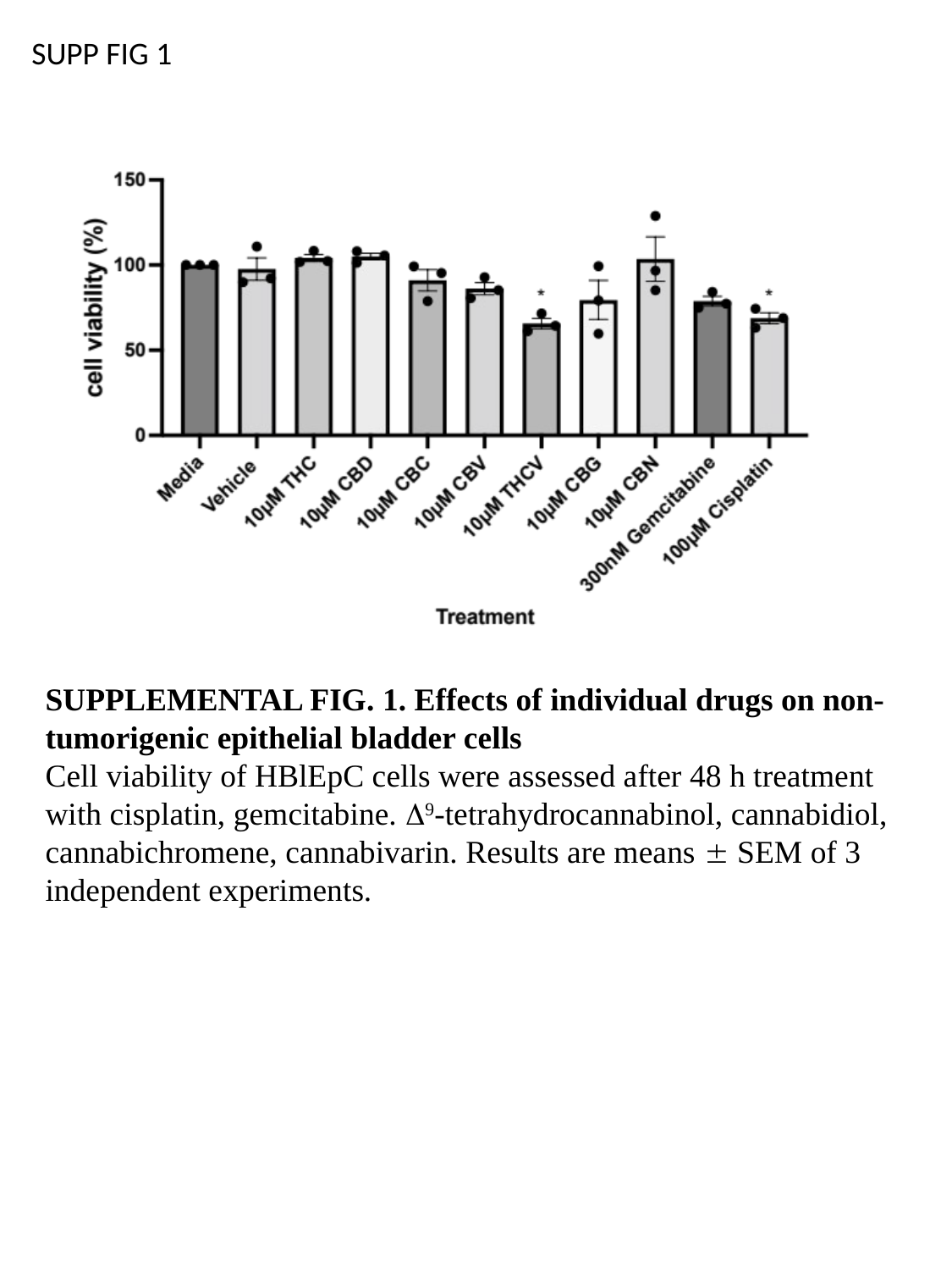

SUPP FIG 1
SUPPLEMENTAL FIG. 1. Effects of individual drugs on non-tumorigenic epithelial bladder cells
Cell viability of HBlEpC cells were assessed after 48 h treatment with cisplatin, gemcitabine. Δ9-tetrahydrocannabinol, cannabidiol, cannabichromene, cannabivarin. Results are means ± SEM of 3 independent experiments.
